# Integrating care in a children’s hospital: a qualitative interview study with mental and physical health professionals in England

**DOI:** 10.1136/bmjopen-2025-113196

**Published:** 2026-03-06

**Authors:** Tessa Morgan, Dihini Pilimatalawwe, Julia Mannes, Isabella Morse, Jessica Folwell, Paula Hain, Catherine M Walsh, Sara O’Curry, Robbie Duschinsky

**Affiliations:** 1 Public Health and Primary Care, University of Cambridge, Cambridge, UK; 2 Mosaic Dance, Cambridge, UK; 3 Liaison Psychiatry Service, Cambridgeshire and Peterborough NHS Foundation Trust, Cambridge, UK; 4 Cambridge University Hospitals NHS Foundation Trust, Cambridge, UK; 5 Cambridge and Peterborough NHS Foundation Trust, Cambridge, UK

**Keywords:** Delivery of Health Care, Integrated, Hospitals, Child, Interprofessional Relations

## Abstract

**Abstract:**

**Objective:**

To explore physical and mental health professionals' hopes and concerns around integrating their services in a colocated children’s hospital.

**Methods:**

One-off semi-structured interviews were conducted with 31 participants (16 mental health and 15 physical health professionals). Participants were purposively sampled to ensure representation across each trust and professional background. Participants included allied health professionals, nurses, team managers, paediatricians, psychiatrists and psychologists.

**Results:**

Staff described integration as both promising and ambiguous. While many welcomed the potential for improved collaboration and holistic care, others expressed uncertainty about what integration would entail. Six key themes were identified: (a) siloed and patchy beginnings, (b) one whole child, (c) day-to-day of colocation, (d) the integrated worker, (e) patients not in the same boat and (f) extending integration.

**Conclusions:**

This novel analysis offers insights around the practical and emotional processes involved in integrating care systems. Staff valued the potential for holistic, child-centred care, improved collaboration and shared learning but expressed concerns around ambiguity, shifting professional identities and the practicalities of colocating services. These tensions underscore the need for clearer communication, relationship-building and support structures during service redesign. Our findings also support integrating wider community and social care systems and call for future research involving young people’s perspectives to ensure meaningful, inclusive integration.

**STRENGTHS AND LIMITATIONS OF THIS STUDY**

- The sample was self-selecting, potentially attracting participants with a particular interest or experience in integrated care, which may introduce response bias.
- Those who chose to participate may differ in perspective from those who did not, possibly affecting the representativeness of the findings.
- The diversity of professional roles strengthens the validity and robustness of the data.
- The study offers only a snapshot in time, as the healthcare system was in flux during data collection and has continued to evolve since.

## Background

 Integrated care has been lauded internationally as a way of responding to the increased complexity of both physical and mental health concerns facing children and adolescents.^1^ The number of children and young people admitted to paediatric wards primarily for mental health problems has dramatically risen.^2^ Emergency departments across the world have noted rises in the frequency of patients attending due to mental health needs.^3 4^ Some examples of hospitalisation that require both mental and physical health attention are eating disorders, self-harm or attempted suicide, drug or alcohol intoxication, or somatisation due to trauma.^5^ There is also evidence that young people who experience chronic or life-limiting physical health conditions, including childhood cancer, are more likely to experience depression and anxiety than their peers.^6 7^ There is a range of calls for new collaborative models to address young people’s interconnected needs and to ensure there is early intervention for mental health distress.^6 8^

There is a growing literature base around the efficacy and implementation outcomes of integrated care models,^9 10^ though the evidence base for children and adolescents remains sparse and of moderate quality. There is tentative evidence that integrative models improve patient satisfaction and perceived quality of care.^9^ There is also evidence that service integration improves young people’s access to physical and mental health care.^11^ For example, one study found introducing child psychiatry in paediatric clinics quadrupled the likelihood of accessing psychiatric treatment in the intervention group.^12^ A meta-analysis found that integrated pathway innovations that included psychological support while in A&E reduced child and adolescent readmissions by up to 37%.^13^

Few studies to date have explored staff experiences of integration, and most of them have centred on quantitative surveys exploring professionals’ confidence and beliefs of delivering integrative care.^14^ We do not know how staff anticipate and experience these changes, particularly in the early phases of design and consultation. Calls have been made for future research on integrated care to prioritise the involvement of staff to ensure their experiences are embedded into and inform future initiatives.^15 16^ At present, there is no gold standard for integrating care.^17 18^ The Royal College of Psychiatry^19^ defines ‘integration’ broadly as making something ‘unified’ or ‘whole’ and ‘where all the parts are brought together to work seamlessly for patients’ (p.4). In health and social care policy, integration tends to be described synonymously with joined-up or coordinated care, typically referring to instances where a range of different services or multiple professionals with different roles within an organisation start working together.^20^ There is growing recognition that you cannot ‘legislate for collaboration’^21^ as the delivery of integrated care ‘ultimately depends on the skills, behaviour and engagement of healthcare workers’ [21, p.1; 22]. Contributing to this literature, this study aims to understand how mental health and physical health staff perceive and imagine integrated care to look in a colocated children’s hospital.

## Methodology

### Study design

This research was commissioned by two NHS trusts in preparation for the development of a new specialist children’s hospital in a metropolitan city in England. This hospital will be the first in the region which serves approximately 1.5 million children. It will include 124 hospital beds: 88 inpatient (72 physical health inpatient beds and 16 PICU) and 36 mental health inpatient beds, 42 day-case beds and seven operating theatres over five storeys. This children’s hospital involves the integration—understood as colocation and joined-up working—of two NHS trusts. Within England, healthcare delivery under the National Health Service (NHS) is organised through NHS trusts, which are regionally based public-sector organisations responsible for providing healthcare services to defined local populations. They operate as non-profit organisations, funded primarily through public taxation.

Currently, children’s services in this area are separated into mental health services delivered by one trust and physical health delivered by another trust. Both trusts currently operate across a range of physical sites. It is important to note that this distinction is somewhat arbitrary as young people entering hospital services often have a range of needs that have both physical and mental health components. Staff in these trusts may also have training in physical and mental health to varying degrees. Figures around how many staff will be impacted directly by the proposed colocation are not publicly available, though in total the two trusts have approximately 16 500 staff (which includes those working with adults and children).

The research itself was conducted by academic researchers independent from the NHS trusts. One experienced qualitative researcher conducted all interviews (TM, female post-doctorate specialised in qualitative methods). The interviewer’s independent status was clearly communicated on participant information sheets and explicitly restated at the beginning of each interview. Some members of the research team were affiliated with one or both trusts. To protect participant confidentiality, only anonymised data was shared with the wider research team. This study received ethics approval from the Cambridge Psychology Research Ethics Committee (Application No: PRE.2022.118)

### Patient and public involvement

Patients and the public were involved in the present study. This children’s hospital project has had sustained patient and public involvement from the start with:

- 6 parent advocates with lived experience sitting at governance level.
- 90 parent carer voice members who meet quarterly but are invited to join workshops based on their specific lived experience.
- 35 young people sitting on Youth and Young Adult Forums who meet monthly to feedback on specific aspects of the project.

The model of integrated care itself came about through engagement with parents, carers, young people and their families with lived experience who are in no doubt about the impact that physical ill health had on a young person’s mental health and vice versa. The initial question for this study was raised as a concern (MH patients on the acute wards) by staff. Parents and carers with relevant lived experience were then brought in to review the preliminary findings with the qualitative researchers as part of the analysis process. Provisional findings of this study were shared in December 2024 by the forum convenors (independent from this study) at the Children’s Hospital Youth and Young Adult Forums. In attendance were 7 Youth Forum members (aged 14–18) and 10 Young Adult Forum members (aged 18–26). The young people’s reflections were made into a report and have been incorporated into the discussion section. We note that young patients were notably aligned in terms of the key areas of hope and concern expressed by professionals.

### Data collection

Recruitment was supported by both NHS trusts. Clinical leads forwarded an email invitation along with the interview questions to inform staff about the study. Participants were invited to take part in a study about their experiences and expectations of integrated working between physical and mental health trusts. Interested staff were required to email the independent researcher to express their interest. To protect participants' confidentiality, no information about who took part was shared with any clinical leads. By taking part, professionals went into a draw for a £300 voucher.

All participants received a copy of the participant information sheet and had opportunities to ask questions about the study before taking part. All participants signed a written consent form before they took part in the research. One-off semi-structured interviews were conducted between November 2023 and March 2024. A total of 31 participants took part, including 16 mental health professionals from one trust and 15 physical health professionals from another (see online supplemental S2 for full sample characteristics table). Mental health professionals belonged to a trust that identified as exclusively providing mental health care and had trained on mental health pathways. Physical health professionals, including occupational therapists, in the second trust had predominantly trained and now focused on providing for children’s physical health needs. As acknowledged below, many of these professionals felt that they also delivered some mental health support because of the high number of mental health presentations in paediatrics. The interviewer, in consultation with the wider research team, felt that sufficient information power was reached with this number of interviews.^22^ Participants were purposively sampled to ensure equal representation across each trust, and a diversity of professional backgrounds was reflected. Participants included allied health professionals, nurses, team managers, paediatricians, psychiatrists and psychologists. Eleven interviews were conducted over Zoom at a time of the participants' choosing. Twenty interviews were conducted in person, including three group interviews consisting of four, five and seven participants, respectively. All three group interviews took place at the group’s place of work. Two members of a group interview decided after the interview that they would not like their audio to be included. The interviewer immediately listened back to the tape to note and redact their comments so that they were not included in the analysis.

All participants were asked the questions stated in the interview guide (see online supplemental S1), which were sent ahead of time to support the transparency of the project. All interviews were audio-recorded and ranged from 10 min to 1 hour. Audio was transcribed verbatim using the online software TRINT. All transcriptions were reviewed and cleaned by the interviewer. One transcript was returned to a participant on request for them to edit sensitive, non-study-related information. All transcripts were anonymised by the interviewer before sharing them with the wider research team.

### Data analysis

We used reflexive thematic analysis which is an inductive, flexible qualitative approach to identifying ‘patterns of shared meaning, cohering around a central concept’.^23^ This method also emphasises the researcher’s active role in knowledge production.^24^ Braun and Clarke^24^ suggest that reflexivity occurs at the intersection of (a) the dataset, (b) the theoretical assumptions of the analysis and (c) the analytical skills/resources of the researcher. Regarding our theoretical assumptions around integration, we understand it as a ‘boundary object’ designed to enable different groups to work together without consensus, rather than an actual concept with a definite meaning.^25^

Once data collection was complete, two researchers familiarised themselves with the data by separately reading through and open coding each of the transcripts. Codes were understood as ‘central organising concepts’ that reflected the researcher’s interpretation of patterns of meaning across the dataset. This process supported our reflexive and thoughtful engagement with the analytic process as it allowed us to ask questions of each other’s interpretations, which helped to clarify meanings. Initial ideas were shared with another senior social science researcher to support the careful clustering of ideas into provision themes.

To promote rigour and reflexivity, these initial themes, originally organised around three hopes and four fears, were presented at three meetings that included professionals and service leads from both NHS trusts. These consultation sessions were a form of member-checking which helped to support the trustworthiness of our interpretation and further contextualise the implications of the findings.^26^ Following this, a team of five researchers worked to further refine categories into the final five themes presented in this paper. These were then shared and further refined through consultation with senior members of each NHS trust. These findings were shared with participants who had the opportunity to provide feedback which further supported their credibility. This analysis aims to capture the diversity of perspectives within each theme. Quotations are presented with detail on whether they were mental health (MH) or physical health staff (PH, which included allied health staff) and where relevant their role.

## Results

Integration of mental health and physical health services into one children’s hospital evoked hopes and fears. On one hand, integration was perceived as a facilitator of new possibilities for greater connection across services, improving communication and streamlining care for young people with interconnected needs. Nevertheless, as integration was a nebulous concept, it meant many participants also expressed anxieties around what kind of change was in store. The concept of integration evoked vulnerabilities, especially where changes were thought to challenge existing professional status and favoured established practices. Imaginaries of integration were heavily shaped by professional status, with mental health staff expressing greater concerns due to the perception it would have a greater impact on their current ways of working. To make sense of integration, participants offered tangible examples relating to their current and imagined future workplace, which we have categorised into the following six themes: (a) the starting point: siloed and patchy; (b) its one whole child; (c) the day-to-day of colocation; (d) the integrated worker; (e) patients won't be in the same boat; and (f) extending integration.

### The starting point: ‘siloed and patchy beginnings’

Staff from both trusts noted that while the children’s hospital was focused on promoting colocation and joined-up working, presently there was little integration between trusts. Professionals from each side identified some forms of ‘siloed and patchy’ integrated care which hinged on “individual teams hav(ing) developed relationships” (MH5). Many participants shared that these professional relationships could develop where professionals with different skill sets were already embedded into the same service (MH5). For example, some mental health staff working in the inpatient unit felt that having an in-house paediatrician meant that most of their patient’ physical health concerns were dealt with in a timely fashion without having to transfer patients to hospital (MH6). Some physical health professionals appreciated working alongside the psychiatry liaison service. As one participant noted, this was facilitated through weekly interprofessional meetings that supported “breaking down barriers and reducing silo working, we know the psych liaison psychiatrist and we know the psychologists and we discuss the cases of barriers between teams” (PH3). Multi-disciplinary team meetings were also identified as improving patient care as these facilitated the “verbal handover” from both sides which “gives a holistic view of the patients” (PH2). These forums were viewed as opportunities to “jointly present cases and there’s a lot of exchange of knowledge and skills” (MH5).

Every participant noted that integrated working was currently inhibited due to trusts having separate computer systems. As one participant clearly put it: “We have got [computer service 1]. And they have [computer service 2]. Well, I think they use [computer service 2]” (PH2). Participants agreed that the limited information sharing between services often resulted in uncertainty around other professionals’ decisions and was sometimes led to delays in care or “duplication of services” (MH1, PH5). The lack of service overlap left a few mental health professionals feeling that they were “treated like an individual who just goes to A&E” rather than as another professional working in the same system (MH 2, 3). As a result, inpatient staff could spend most of their shift waiting with a patient to be seen at (the hospital) (MH 2,3), resulting in the mental health inpatient unit being short staffed. A handful of professionals shared the work-arounds they had developed to overcome barriers to information sharing that included taking photographs of notes and manually uploading them (PH1) or else asking families for additional information from primary care (MH4).

### “It is one whole child”

The principal hope for the new children’s hospital was that it would promote a culture shift towards holistic and child-centred care. This was underpinned by the perspective that siloed working resulted in arbitrary separation of young people’s needs:

I've always wanted to break down mental and physical health because it is one child, it is one whole child, one family, it’s one unit. So I think the vision is absolutely fantastic (PH5)

For health professionals, the need for more holistic care was shaped by the growing number of patients with mental health needs appearing on their wards. Overdoses and eating disorders were identified as increasingly common reasons for admissions to A&E, where the root cause was thought to be mental health related. One health professional with managerial responsibilities noted that integrating care was “a really good and noble idea” especially because:

mental health is creeping into physical health so much more and there are times when the majority of our patients are mental health patients which is interesting. And even if you've got physical problems, there’s mental health there…there is some provision thank goodness. But it'd be nice for that to be more integrated into the patient stay (PH1).

Many participants expressed that a dual approach would be particularly suited to the inter-related mental and physical health needs of eating disorder patients. It was also felt that the mental health needs of patients with comorbid, complex physical health needs, such as cystic fibrosis, could be better ‘picked up’ (PH9) as a result of colocation. For example, one professional working on a paediatrics ward reflected:

I do think some people probably do end up slipping through, the long term patients or people who have got like chronic health conditions, who are in and out, perhaps don't stay in for a long time, but they are like, say for example, cystic fibrosis patients who they come for about 2 weeks stints and then they're gone. I think acknowledging that those sorts of patients, I think (mental health) would be picked up a lot more (PH9).

Many participants also felt that the new hospital offered an opportunity to design a space that is better tailored to children’s “special needs, special services” (MH4). There were hopes of including more children-sized equipment including toilets, cutlery, bathing and gym facilities. An occupational therapist hoped the protected space for children would improve patients’ experience during their stay:

So it will be nice to be in a protected space for children. I think for that reason, hopefully even just the graphics of the paint and the activities and things will be more specific to children so they can hopefully make it look a bit less like a scary clinical hospital (PH7).

### The day-to-day of colocation

Most participants felt that colocation would improve the day-to-day relationships and communication between professionals which would in turn improve care delivery. Physical health professionals in particular felt that “working from the same hubs” could support greater multi-disciplinary working that would be “more timely and less silo working” (PH6). Professionals shared aspirations for quicker referrals, discharges and safer transfers between locations. Multiple participants expressed that being able to “knock on someone’s door and speak to the people right there face-to-face” could help expedite care decisions (PH1) and enable more holistic care plans (MH5). A few outliers felt that due to the use of online meetings, “being on site isn’t as important as it used to be” (MH5).

Participants also expressed concerns about operational challenges posed by colocation. Both physical and mental health professionals catalogued instances where the “separate ways of working” (PH6) between the trusts would likely result in conflict or challenges, at least in the early phases, of the new hospital. Participants shared their confusion around who the hospital would serve given that, as with most NHS trusts, “(mental health) will have to go to 18. Paediatrics to 16” (MH5). Concerns were raised about the different pace of working given the turnover of patients was a few days in physical health vs a few months in mental health. Staff also raised questions about what would happen with uniforms which symbolically distinguished mental health and physical health nurses, as one nurse manager put it:

historically mental health nurses have never worn uniform. Physical health nurses have worn uniform. I mean, I tried to get away from that because I think we're all nurses…But culturally there is a difference there” (PH5).

Other logistical questions frequently raised by members of both trusts were about

whether the trusts would be merging and if so, who would be paying their salaries or to whom would they request leave from. Notably, some of these uncertainties arose from different staff having different kinds of information about the new hospital, with some participants being involved in planning and consultation, whereas some were “getting their information from the newspaper”(PH1).

Staff frequently discussed the opportunity cost of integration, lamenting the loss of currently valued aspects of their separate services. Physical health professionals frequently raised concerns that some of the children’s services currently colocated in the hospital would be fragmented. For example, whereas “paediatric intensive care is moving… the children’s A&E is not moving, the children’s outpatients is not moving”. It was felt that this would impede timely care because rather than the Paediatric Intensive Care Unit being two floors away “at (the) new children’s hospital they will be about two kilometres away from resus” (PH3). Physical health professionals also raised concerns about changes to their office spaces and car park availability with worries that the new hospital would not be able to accommodate everyone.

For mental health professionals, the change was felt keenly by those who would be shifting from another site to the new hospital. For some, there were fears that this shift in location would also be linked with a shift away from their valued “family model of care” (MH6) towards a more “biomedical” model (MH1). In particular, mental health professionals shared concerns around whether parents would still be able to be admitted alongside their child, which was viewed as a key pillar of their current care model. Participants in the group interview collectively expressed sadness about the loss of the “beautiful green space” surrounding their mental health facility which offered “a nice quiet environment” for the young people under their care and a chance for them to develop their independence as part of their recovery:

That’s really nice for patients and parents, you know, and you know they can just go out and go for a walk down to the village or they can walk to Tesco, you know, they can get on the bus. Everything’s really accessible. But it’s a nice quiet environment (MH6).

### The integrated worker

Most physical health professionals viewed the new children’s hospital as an opportunity to develop their skills needed in their current roles. Many acknowledged that because they had chosen to specialise in physical health, they felt they had not received “in depth” training around mental health. Instead, their knowledge and skills around mental health were “experience based” from encountering mental health patients on the wards (PH 5, 9, 10). Physical health staff noted that currently there was a lot of professional anxiety when interacting with patients with mental health concerns. Integrated care was therefore viewed as a welcomed opportunity for professional and skill development. Multiple student nurses recognised that it would be helpful to learn how to communicate with “eating disorder patients around mealtime and people about having some sort of mental health crisis, would be really good” (PH9). By contrast, all allied health professionals interviewed perceived their roles as inherently holistic as they supported young people’s mental and physical health. Rather than learning new skills, they shared hopes that the new hospital would present an opportunity for a greater recognition of their skillset and more opportunities for them to improve young people’s well-being through greater input for physiotherapy, speech and language therapy for a wider patient population.

Many mental health professionals expressed concerns that relocating to a hospital threatened their professional identities. Multiple mental health professionals reiterated that they had chosen to specialise in mental health precisely because they “don't want to do physical. They just want to do mental health”(MH6). The potential dissolution of this professional distinction and being “mixed across physical and mental health” was described as causing much “anxiety” (MH6). Mental health professionals asserted that it was not comparable kinds of work as one participant outlined:

And now you might even have to move from physical to mental. Then 1 day mental to physical. You can't pour from an empty cup though. I mean, also for us we have to be mentally stable, you know? (MH2)

This was set within wider concerns that “it’s probably not going to be as smooth” given that mental health professionals already felt some “animosity” directed towards them in situations where they were required to “take over” the care of their mental health patient in an A&E setting (MH6). Some mental health professionals also perceived that physical health professionals sometimes placed unrealistic expectations on them to resolve their patients’ difficulties.

### Patients not in the same boat

Professionals raised concerns about potential challenges to bringing mental health and physical health patients together. While multiple physical health professionals expressed a sense that the integration of mental health and physical healthcare would help to normalise mental health difficulties, as one staff member put it: “you want some integration and some normalisation and that everybody has mental health and we have to look after it” (PH6). On the other hand, many provided examples where patients “won’t be in the same boat” to highlight how their needs were not commensurate. For example, “one child might not be allowed to eat because they were pre-op vs eating disorder patients who were refusing to eat” (PH10). In another instance, a mental health professional acknowledged that “if you have somebody who’s really physically unwell and somebody screaming, “I want to die”. It’s not very sensitive” (MH1). Many also drew on examples of encounters on physical health wards where mental health patients were perceived to exhibit challenging and disruptive behaviour that was unsettling for patients and their families. Professionals offered examples of how it was “not very nice” to have to witness “screaming or self-harming”(MH1).

A range of professionals also expressed concerns about the impact of integration on mental health patients’ care. There were concerns that shifting mental health patients into a space that “feels more medicalised” may “complicate their perception of themselves and their treatment” (MH6). This was reinforced by patients’ reflections that they already felt stigmatised, as one professional shared that her patient “felt like people were looking at her because she was obviously from a Psych ward” (MH6). Multiple references were made regarding the concerns about the implications on mental health patients’ dignity in instances where they tried to abscond and then were returned in restraints, as one mental health practitioner stated, “the child’s dignity will be out the window because everyone will see it” (MH6).

### Extending integration

Both sets of professionals expressed an interest in expanding the remit of integration to account for the multiplicity of young people’s needs entering their services. There was a particular interest in social care playing a greater role in the new hospital given that many young people with interconnected physical and mental health needs often had delayed discharges due to challenges accessing support for their “social situation” (PH1, 13):

You know, we need to integrate in lots of different places, education, with social care. You know just there’s so many kind of agencies involved now and other places of integration that need serious attention (MH5)

There was also enthusiasm for more integration with community care. Some participants explicitly acknowledged that there was some promise of promoting integration with community services, epitomised in the slogan “Hospital Without Walls”(MH5). There were nevertheless concerns that “we haven't seen that on the ground” (MH5). Such scepticism was reinforced by the current perceived limited integration between community CAMHS and outpatient liaison services. Greater integration between hospital-based and community services was viewed as being in line with “paediatric philosophies …always trying to keep our children at home” (PH5). This goal was also raised by mental health professionals who also acknowledged that “the majority of our children never, ever reach an inpatient ward, and that is the aim in any case, they never reach an inpatient ward” (MH5). Integration therefore offered an opportunity for thinking about how young patients in the community could be included further in the new hospital designs.

## Discussion

This study offers an important addition to the emerging literature around integrated care by exploring the experiences of mental and physical professional staff during the transition towards an integrated children’s service. Building on previous studies, this study illustrates the importance of capturing professionals’ perspectives through the process of service redesign in order to develop a multifaceted understanding of how these changes were interpreted and imagined by those expected to deliver such care.^15 27^ This study first identified key aspirations staff had for integrative care. Mirroring evidence in the literature, our findings indicate that professionals acknowledged that young people had interconnected mental health and physical health needs which could be better met with better coordination of care between respective services.^1^ Integration was viewed as an opportunity for developing child-friendly services that acknowledged the holistic and unique needs of children and young people. As identified in previous studies, colocation was viewed as a means to improve communication, especially as it afforded opportunities to build face-to-face relationships between professionals with different skill sets.^28^ Similar to previous findings, participants in this study prioritised the integration of computer systems to improve their joined-up care provision.^19 29^ As noted in previous studies,^15 30^ integration was also viewed as an opportunity for further learning and knowledge exchange, most notably for physical health staff around mental health skills.^15 31^ This supports calls made by NHS England for appropriate mental healthcare to be made available in physical healthcare settings.^32^

Our second main finding is that because integration was such an ambiguous concept, it produced uncertainties and anxiety among staff. Staff were particularly concerned about changes to workplace culture and their professional identities. This analysis supports previous findings that service leads need to provide clear definitions of what integration means early in the process, with our analysis suggesting particular attention be paid to implications for workplace culture and professional identities^11^ as well as consideration of broader organisational arrangements including leadership and pay structures. In line with previous research, we identified that all of our participants sought clarity over their roles,^16^ something that has been identified as particularly important where integration involves the colocation of services.^16^ There was also a shared sense that it would take time to adjust to change, which is something echoed in studies with staff experiences of integrating their services [3441].^33^

Our analysis indicates that part of this adjustment will involve facing the loss of some beloved aspects of previous service delivery. This finding amplifies previous findings around the importance of addressing unexpected consequences of change^29^ and clearly acknowledging and communicating the reasons behind trade-offs. Taken together, these findings are important considering evidence that successful integrative initiatives rely on sustained support from staff^21 34 35^ and that workplace satisfaction connects with improvements in healthcare provision.^36^ We also identified positive examples where integration was already working, which revolved around opportunities for professionals to build relationships with other professionals, for example, liaison psychiatry or multi-disciplinary team meetings. Through further consultation sessions, professionals highlighted how arranging exchange programmes between physical and mental health teams prior to colocation may be a useful strategy to help build relationships between services.

A third key finding was about professionals’ perceptions of the challenges involved in bringing mental health and physical health populations together. Most of the research has tentatively posited that integrative care will improve patient outcomes as well as access to services.^11^ Professionals in this study focused predominantly on the potential challenges of bringing physical and mental health populations together given their different needs and perceptions of challenging behaviours of mental health patients. The findings about fears of the intensification of stigma around mental health patients due to close proximity are a concern shared by other mental health academics.^37^

Interestingly, participants in the Children’s Hospital Youth and Youth Adult Forums expressed concern about all patients being mixed in and rather endorsed a tier system where “really poorly mental health patients should be put somewhere away from the less unwell mental health patients.” Yet this forum agreed that physical and mental health needs should be taken care of “at the same time so that you leave hospital feeling better both physically and mentally”.

Our recommendation is that more efforts need to be taken to promote a ‘parity of esteem’ between mental and physical health services in order to achieve effective joined-up care.^19 37^ Services will need to explicitly address any pre-existing power imbalances between mental and physical health professionals. We suggest that there could be a benefit to facilitating explicit forums around power sharing between the services as well as the opportunity for exchange programmes so that mental and physical health staff can experience their counterparts' roles and responsibilities prior to their colocation. Future research is required with child and adolescent patients to gain understanding of their hopes and apprehensions of integrated care, especially around how to avoid entrenching stigma and ensure their views and solutions are incorporated into service delivery plans.^38^

A fourth key finding was that effective joined-up working needs to go beyond colocation in a hospital and be embedded in wider community and outpatient services. The Royal College of Physicians^39^ has similarly endorsed this, given that the majority of young people’s mental and physical health needs are not treated in hospitals but at home through outpatient support. A key recommendation emerging from the consultation process on this manuscript was that young people with eating disorders are a priority group who will require integration between community-based services and the new hospital. Our findings also identify a need for greater integration with children’s social care as a result of the complex nature of young people’s presentations. This finding is supported by previous studies including evidence^40^ that those who frequently attended A&E had complex mental and social health profiles.^4^ This finding was strongly endorsed by the Children’s Hospital Youth and Youth Adult Forum, with one participant noting, “we need more of a link with social care as many children have issues as a result of their social situation”. Joining up care with social care is in line with NHS England guidance that integrated systems ought to consider the wider determinants of health when setting the overall direction of the new service.^41^

### Limitations

One limitation of this study is that this is a self-selecting sample, which may have led to the participation of individuals with a particular interest or experience. This could introduce a degree of response bias, as those who chose to engage in the interviews may differ from those who did not. However, we feel that the diversity of perspectives, including a range of professionals across different roles and settings, helped to strengthen the robustness and validity of our findings.

Second, this study and analysis is able to only capture a snapshot of a healthcare system in transition, as changes have occurred since the data was initially collected. Influenced in part by early findings shared with the trusts, procedural and logistical changes have already occurred. As a result, the uncertainties and anxieties reported by participants may have evolved or been mitigated through new policies, training and additional information about plans for integrating the new children’s hospital. We recommend further longitudinal mixed-method research to gain further understanding of changes in professional understanding and experiences of integration across the period of implementation.

## Conclusions

This study offers an important addition to the emerging literature around integrated care by exploring the experiences of mental and physical professional staff during the transition towards an integrated children’s service. Building on previous studies, this study illustrates the importance of capturing professionals’ perspectives through the process of service redesign in order to develop a multifaceted understanding of how these changes were interpreted and imagined by those expected to deliver such care. This study outlines the implication of integration of professionals’ views on their working culture, on their professional identities and outcomes for patients. We conclude with implications for policy and practice, including the need to strengthen professional relationships and shared computer systems, to consider power imbalances between professionals. We also identified the potential need to extend integrative efforts to include community care provision and social care in order to best attend to the interconnected needs of young people.

## Supplementary material

10.1136/bmjopen-2025-113196 online supplemental file 1

10.1136/bmjopen-2025-113196 online supplemental file 2

## Data Availability

No data are available.
