# Supplementary material for: Integrating care in a children’s hospital: a qualitative interview study with mental and physical health professionals in England
Source: BMJ Open. 2026 Mar 6;16(3):e113196. doi: 10.1136/bmjopen-2025-113196 (PMC12970107; doi:10.1136/bmjopen-2025-113196)
Supplement: online supplemental file 1 [file bmjopen-16-3-s001.pdf]

S1: *Interview guide with prompts for*

1. In your current role, do you interact with trust1 / trust2 staff?
2. What is the nature of those interactions (eg. clinical, managerial, on-call, etc)?
3. What is your experience of those interactions?
    a. What is positive?
    b. What could be better?
4. How would you hope to interact/work together in the Children's Hospital?
