# Supplementary material for: Integrating care in a children’s hospital: a qualitative interview study with mental and physical health professionals in England
Source: BMJ Open. 2026 Mar 6;16(3):e113196. doi: 10.1136/bmjopen-2025-113196 (PMC12970107; doi:10.1136/bmjopen-2025-113196)
Supplement: online supplemental file 2 [file bmjopen-16-3-s002.pdf]

S2: *Table of Sample characteristics of Professionals Interviewed*

Gender		n
	Female	28
	Male	3
Physical Health Roles		
	Paediatrician	3
	Nurses and Health care assistants	5
	Managers	2
	Allied Health Care Professionals	5
Mental Health Roles		
	Nurse and Health care assistants	7
	Psychologists	3
	Psychiatrist	4
	Managers	1
	Community CAMHS	1
