# Supplementary material for: Integrating care in a children’s hospital: a qualitative interview study with mental and physical health professionals in England
Source: BMJ Open. 2026 Mar 6;16(3):e113196. doi: 10.1136/bmjopen-2025-113196 (PMC12970107; doi:10.1136/bmjopen-2025-113196)
Supplement: Supplementary file 3 [file bmjopen-2025-113196.reviewer_comments.pdf]

PEER REVIEW HISTORY

BMJ Open publishes all reviews undertaken for accepted manuscripts. Reviewers are asked to complete a checklist review form and are provided with free text boxes to elaborate on their assessment. These free text comments are reproduced below.

ARTICLE DETAILS

Title (Provisional)

Integrating care in a Children's Hospital: a qualitative interview study with mental and physical health professionals in England

Authors

Morgan, Tessa; Pilimatalawwe, Dihini; Mannes, Julia; Morse, Isabella; Folwell , Jessica; Hain, Paula; Walsh, Catherine M.; O'Curry, Sara; Duschinsky, Robbie

---

VERSION 1 - REVIEW

**Reviewer**	**1**
**Name**	**Reilly, Rachel**
**Affiliation**	**South Australian Health and Medical Research Institute, Wardliparingga**
**Date**	**28-Nov-2025**
**COI**	**None**

Thanks for the opportunity to review this paper. It is an enjoyable read and addresses an important topic. Including the views and experiences of service providers working within systems that are undergoing significant change is important for managing the change effectively for all parties and mitigating risks. My comments are below.

Introduction

1) As someone based outside of England, I think it may be helpful to include 'in England' in the title.

2) Similarly, given this paper relates to a very specific case of systems change in the development of a new hospital, some of the issues raised may be context dependent. Therefore, richer information about the context in which this is taking place would be useful. For example, is this a regional or metropolitan hospital? How many staff will be affected? What does the current non-integrated care look like?

3) Also, a description of 'trusts' – what are they and what do they do? (in both health care delivery and this research). Trusts may not be familiar to readers outside the UK.

4) Integrated care is defined in quite vague terms at the end of the introduction, and then later as a 'boundary object'. Whilst acknowledging that there may not be a clear definition in the literature, related to the above comments, it would be helpful to provide a definition earlier in the paper that is relevant in this context– ie what do the trusts who are developing the hospital mean by integrated care?

Patient and Public Involvement

5) I'm not sure that feedback from practitioners on findings meets the definition of 'Patient and Public Involvement,' as outlined by BMJ: https://www.bmj.com/sites/default/files/attachments/resources/2018/03/PPI_in_Research.pdf

Some more detail about how patients were involved in setting the research priorities would strengthen this section.

Data Collection

6) The participants are divided into 'mental health professionals' and 'physical health professionals' and represent a mix of professions. Is the definition of a participant as 'MH' or 'PH' made according to the patients they work with or their professional background? As a general comment, I think it would be worth acknowledging that this is something of a false dichotomy – mental and physical health do not exist in isolation from one another. It could be informative to have a table of participants showing which have been placed in which category.

7) It is curious that two participants withdrew after the interview. Why was this? If there was no reason given (which is fine) this could be stated.

8) Curious about the group interviews. Why was the decision made to conduct both individual and group interviews? Were the group interviews different methodologically? Would being in a group have influenced the findings? How is a group interview with seven participants distinguished from a focus group?

9) The interview guide is very short. It would be good to know what other prompts the interviewer used to elicit more information.

10) 10 minutes to one hour is quite a range – what happened in the 10-minute interviews?

Results

11) I found that some quotes were more like fragments than useful illustrations of the themes. Whilst the use of single words or phrases included in the author's paraphrasing is good for readability, I would suggest that this should be balanced with some more quotes that are illustrative examples of the themes and provide insight into the authentic voices of the participants.

12) Starting point "siloed and patchy" – could the authors clarify whether the participants are commenting on the current level of integrated work ie – is the 'starting point' before the new hospital is developed, and integration formally occurs?

Discussion

13) On p.19 the authors state 'Our final finding was that professionals problematised he boundaries of integrated care.' – this is an interesting point that does not seem to be in the findings themselves. Could the authors link this idea and its implications more clearly to the findings (eg. perhaps offer an example).

Minor comments

14) the quote included in the introduction, "where all the parts are brought together to work seamlessly for patients" - as a direct quote, requires a page number).

15) P.9, last line 'was sometimes attributed to…' seems incorrect- should this be '…sometimes led to'?

16) p.18: last line: I think 'the centrality of facilitating' can be deleted.

**Reviewer**	**2**
**Name**	**Okolo, Anene**
**Affiliation**	**Federal Medical Center, Asaba, Pediatrics**
**Date**	**24-Dec-2025**
**COI**	**None**

This is a well-conceptualised work. It is quite inspiring

VERSION 1 - AUTHOR RESPONSE

Comment from	Comment	Author's response
Reviewer: 1	Thanks for the opportunity to review this paper. It is an enjoyable read and addresses an important topic. Including the views and experiences of service providers working within systems that are undergoing significant change is important for managing the change effectively for all parties and mitigating risks. My comments are below.	Thank you for the kind words and deep engagement with our work.
Reviewer: 1	As someone based outside of England, I think it may be helpful to include 'in England' in the title.	We have amended our title in the article and submission portal to now read as: Integrating care in a Children's Hospital: a qualitative interview study with mental and physical health professionals in England
Reviewer: 1	Similarly, given this paper relates to a very specific case of systems change in the development of a new hospital, some of the issues raised may be context dependent. Therefore, richer information about the	A more in-depth description of publicly available information has been added to page 5. This hopefully illustrates the significance of the hospital as it will be the central hub for 1.5 million children in the region.

	context in which this is taking place would be useful. For example, is this a regional or metropolitan hospital? How many staff will be affected? What does the current non-integrated care look like?	
Reviewer: 1	Also, a description of 'trusts' – what are they and what do they do? (in both health care delivery and this research). Trusts may not be familiar to readers outside the UK.	We have added a definition of trusts on page 5 in line with this definition: https://www.legislation.gov.uk/ukpga/2003/43/notes/division/5/1?view=plain
Reviewer: 1	Integrated care is defined in quite vague terms at the end of the introduction, and then later as a 'boundary object'. Whilst acknowledging that there may not be a clear definition in the literature, related to the above comments, it would be helpful to provide a definition earlier in the paper that is relevant in this context– ie what do the trusts who are developing the hospital mean by integrated care?	Integration in this context means both colocation bringing two services that were previously separate together into one space and attempting to join-up care (albeit the parameters of this are currently blurry). This working definition has been added on page 5 to hopefully make it clearer what we mean by integration in this study.
Reviewer: 1	Patient and Public Involvement<br>I'm not sure that feedback from practitioners on findings meets the definition of 'Patient and Public Involvement,' as outlined by BMJ: https://www.bmj.com/sites/default/files/attachments/resources/2018/03/PPI_in_Research.pdf Some more detail about how patients were involved in setting the research priorities would strengthen this section.	In consultation with NHS collaborators we have re-written this section to reflect the far-reaching role patients and carers have played throughout the whole Children's Hospital project and specifically for this study on page 6.
Reviewer: 1	The participants are divided into 'mental health professionals' and 'physical health professionals' and represent a mix of professions. Is the definition of a participant as 'MH' or 'PH' made according to the patients they work with or their professional background?	A further elaboration has been offered on page 7. In summary:<br><br>Mental health professionals belonged to a trust that identified as exclusively providing mental health care and had trained on mental health pathways. Physical health professionals, including occupational therapists, in the second trust had predominantly trained and focused on providing for children's physical health needs. As acknowledged below many of these professionals felt that they also delivered some mental health support because of the high number of mental health presentations in paediatrics.
Reviewer: 1	As a general comment, I think it would be worth acknowledging that this is something of a false dichotomy – mental and physical health do not exist in isolation from one another.	We completely agree that there is a false dichotomy between mental and physical health. We have noted the porous nature of this both in terms of patient needs and professionals' skillsets on page 5 and have woven this point in again on page 7.
Reviewer: 1	It could be informative to have a table of participants showing which have been placed in which category.	We have added a table as part of the supplementary files that provides more detail about which participants fall into which category
Reviewer: 1	It is curious that two participants withdrew after the interview. Why was this? If there was no reason given (which is fine) this could be stated.	We agree it was an interesting dimension of the research process which we felt we should document for transparency. The two participants who withdrew were part of a group interview of 5 other professionals from their service. One person who withdrew after the interview (note she had initially signed her consent form) expressed her frustrations around the change in their service (which meant she was going to quit). She said she didn't want to be involved in the restructure and then at the end said she'd actually rather not be part of this research as it was connected. Another

		participant handed the consent form quietly back to the researcher blank to indicate he did not want to take part. In both cases the interviewer's sense was that what underpinned these instances of withdrawal was frustration with the changes in the service rather than issues with the research process itself. Out of respect for their wishes, these individuals' contributions were redacted and not included in the analysis process. To do so the interviewer immediately transcribed the audio using TRINT software and redacted comments.
Reviewer: 1	Curious about the group interviews. Why was the decision made to conduct both individual and group interviews? Were the group interviews different methodologically? Would being in a group have influenced the findings? How is a group interview with seven participants distinguished from a focus group?	The initial project was developed on the basis of individual interviews but flexibility was built into the ethics application just in case. Group interviews were requested by the three teams so that as a team they could discuss the Children's Hospital given it will shortly impact their day-to-day service provision. Practically it also worked in terms of timing (e.g. at the start of a shift and in a lunch break). We used the phrase group interview as the interview schedule did not differ from the individual interviews. Unlike a focus group analysis we did not analyse the interactions between participants but treated data as one would analyse an individual interview (in terms of thinking about participants discretely). It is an approach we have used before in another study that was reliant on convenience sampling (e.g who we could get to speak to us): DOI: 10.1017/S0144686X1900165X
Reviewer: 1	The interview guide is very short. It would be good to know what other prompts the interviewer used to elicit more information.	The interview questions are listed below (slight more expanded than what was included in the manuscript). We have included the full set in the manuscript as a supplementary file. Prompts involved asking further questions if participants identified an aspect of their experience, or a particular hope or concern e.g. "could you tell me more about …"<br><br>Interview guide:<br>- In your current role, do you interact with trust1 / trust2 staff?<br>- What is the nature of those interactions (eg. clinical, managerial, on-call, etc)?<br>- What is your experience of those interactions?<br>- What is positive?<br>- What could be better?<br>- How would you hope to interact/work together in the Children's Hospital?
Reviewer: 1	10 minutes to one hour is quite a range – what happened in the 10-minute interviews?<br>Results	The 10 minute interviews were with student nurses on their lunch breaks. We were able to go through the interview schedule in this time as these two student nurses had had a) limited training in mental health provision and b) limited experience of directly supporting young people's mental health. They both expressed they really would like more training as they were quite nervous when they had encountered mental health patients previously (with other staff members typically taking over).
Reviewer: 1	I found that some quotes were more like fragments than useful illustrations of the themes. Whilst the use of single words or phrases included in the author's paraphrasing is good for readability, I would suggest	Thank you for this. We have taken this as an opportunity to expand on the quotes included in the results in order to showcase the richness of the data.

	that this should be balanced with some more quotes that are illustrative examples of the themes and provide insight into the authentic voices of the participants.	
Reviewer: 1	Starting point “siloed and patchy” – could the authors clarify whether the participants are commenting on the current level of integrated work ie – is the ‘starting point’ before the new hospital is developed, and integration formally occurs?	Siloed and patchy refers to professionals’ perspectives of the unevenness of integration – understood as joined-up care and colocation – in the present system. An additional introductory sentence has been added to clarify on page 10.
Reviewer: 1	On p.19 the authors state ‘Our final finding was that professionals problematised the boundaries of integrated care.’ – this is an interesting point that does not seem to be in the findings themselves. Could the authors link this idea and its implications more clearly to the findings (eg. perhaps offer an example).`	Thank you for drawing attention to this. We have clarified what we intended by this paragraph which was to highlight a “fourth key finding that effective joined up working needs to go beyond colocation in a hospital and be embedded in wider community and outpatient services.”
Reviewer: 1	the quote included in the introduction, “where all the parts are brought together to work seamlessly for patients” - as a direct quote, requires a page number).	A page number has been added
Reviewer: 1	P.9, last line ‘was sometimes attributed to…’ seems incorrect- should this be ‘…sometimes led to’?	This line has been checked and amended to be grammatically correct.
Reviewer: 1	p.18: last line: I think ‘the centrality of facilitating’ can be deleted.	This sentence fragment has been removed as we agree that it was unnecessary repetition
Reviewer 2	This is a well-conceptualised work. It is quite inspiring	Thank you for your kind words.

VERSION 2 - REVIEW

**Reviewer**	**1**
**Name**	**Reilly, Rachel**
**Affiliation**	**South Australian Health and Medical Research Institute, Wardliparingga**
**Date**	**16-Feb-2026**
**COI**	

I appreciate the authors thoughtful responses to my queries and am happy to recommend the work for publication.
